# Supplementary material for: Anti-Cancer Activity of Verteporfin in Cholangiocarcinoma
Source: Cancers (Basel). 2023 Apr 25;15(9):2454. doi: 10.3390/cancers15092454 (PMC10177077; doi:10.3390/cancers15092454)
Supplement: Supplementary file 1 [file cancers-15-02454-s001.zip › Supplement figure legends and tables.pdf]

**Supplement figure S1. Schema of treatment experiment plan**

**Supplement figure S2.** (S2A) The expression levels of YAP1 signaling downstream targets in cholangiocarcinoma cohort in TCGA cancers and the corresponding normal tissues. (S2B) correlation between representative genes of YAP1 signaling downstream targets with SOX9. (S2C) Correlation between the expression of representative individual of YAP1 signaling downstream targets with immune suppressive cell infiltration. *p-value < 0.05; **: p-value <0.01; ***: p-value <0.001.

**Supplement Figure S3. Immune cell populations of YAP/AKT model**

C57BL/6J mice were assigned into two groups (n =6/each group), vehicle or verteporfin (VP) was treated every three days three weeks after YAP/AKT plasmids injection. Liver samples were harvested and processed for flow cytometry (S3A) frequency (%) of B cells, dendritic cells (DC), CD4, CD8 and macrophages. (S3B) frequency (%) of mMDSCs and DC in F4/80- frequency (%) = proportion (%) of each parent cells as indicated Y axis. Values are means ± SD.

**Supplement Figure S4. Flow cytometry gating strategy** for (S4A) immune profiling, (S4B) T cell activation/exhaustion and (S4C) myeloids panels.

**Supplement Figure S5. Single-cell analysis of liver and CCA from YAP/AKT mice treated with vehicle and verteporfin, respectively.** (S5A) Violin plot showed specific gene expression of cell type-specific markers. (S5B) The individual gene UMAP plots showing the expression levels and distribution of representative markers of known cell types from the liver tissue/tumors from YAP/AKT CCA mice. (S5C-S5E) Comparison of proportion of TAM-M2, B and CD8 T cells between vehicle and verteporfin treatment group.

**Supplement Figure S6.** Single-cell analysis and comparison of subset of CD8 T cells (A), CD4 T cells (B), and TAMs (C) from YAP/AKT mice treated with verteporfin and vehicle.

**Supplement Figure S7. Volcano plot showed the differential expressed genes of malignant cells from YAP/AKT mice treated with verteporfin in comparison to vehicle.**

**Supplement Figure S8.** GSEA analysis of upregulated pathway in malignant cells with verteporfin treatment.

**Supplemental Table S1. Cell counts from single cell RNA sequencing.**

**Supplemental Table S2. GSEA analysis of all differential expressed genes in all cells between treated with verteporfin and vehicle.**

**Supplemental Table S3. GSEA analysis of all differential expressed genes in malignant cells between treated with verteporfin and vehicle.**
