# Supplementary material for: Anti-Cancer Activity of Verteporfin in Cholangiocarcinoma
Source: Cancers (Basel). 2023 Apr 25;15(9):2454. doi: 10.3390/cancers15092454 (PMC10177077; doi:10.3390/cancers15092454)

**Supplemental Figure S1**

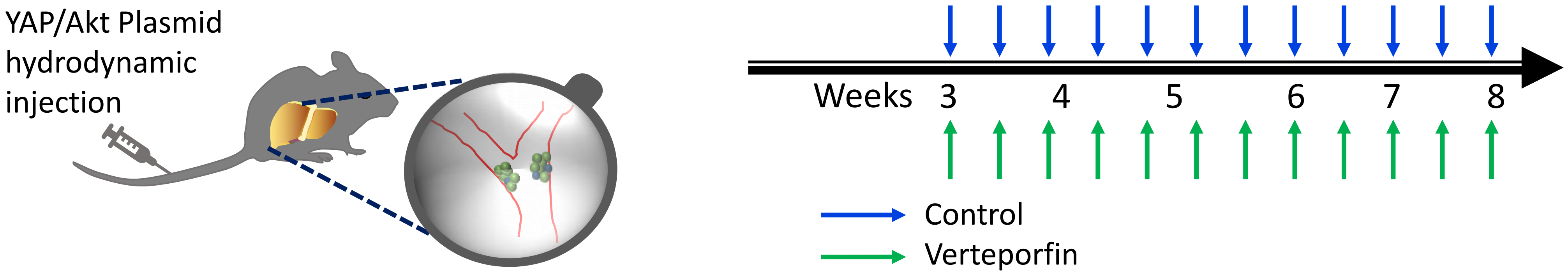

# Supplemental Figure S2

## S2A

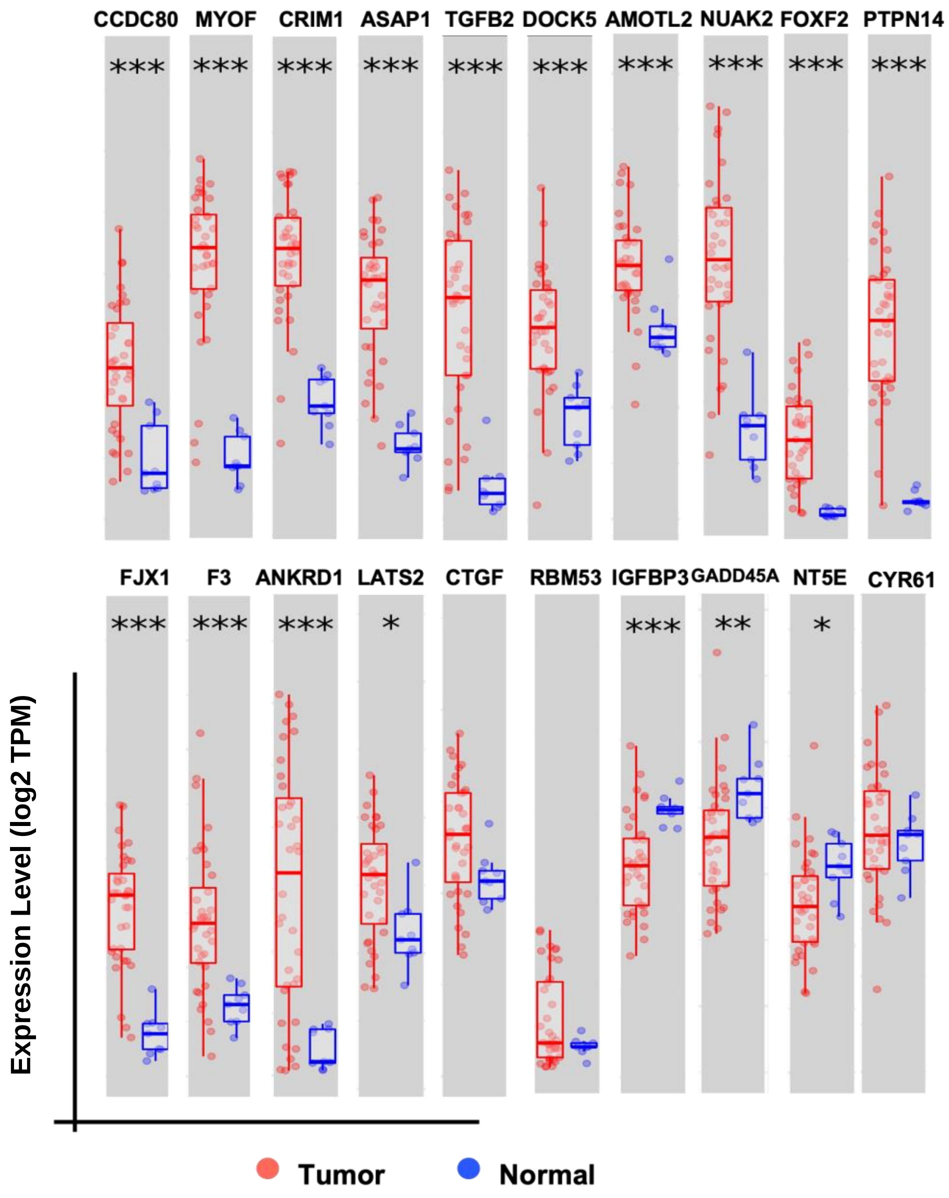

# Supplemental Figure S2

## S2B

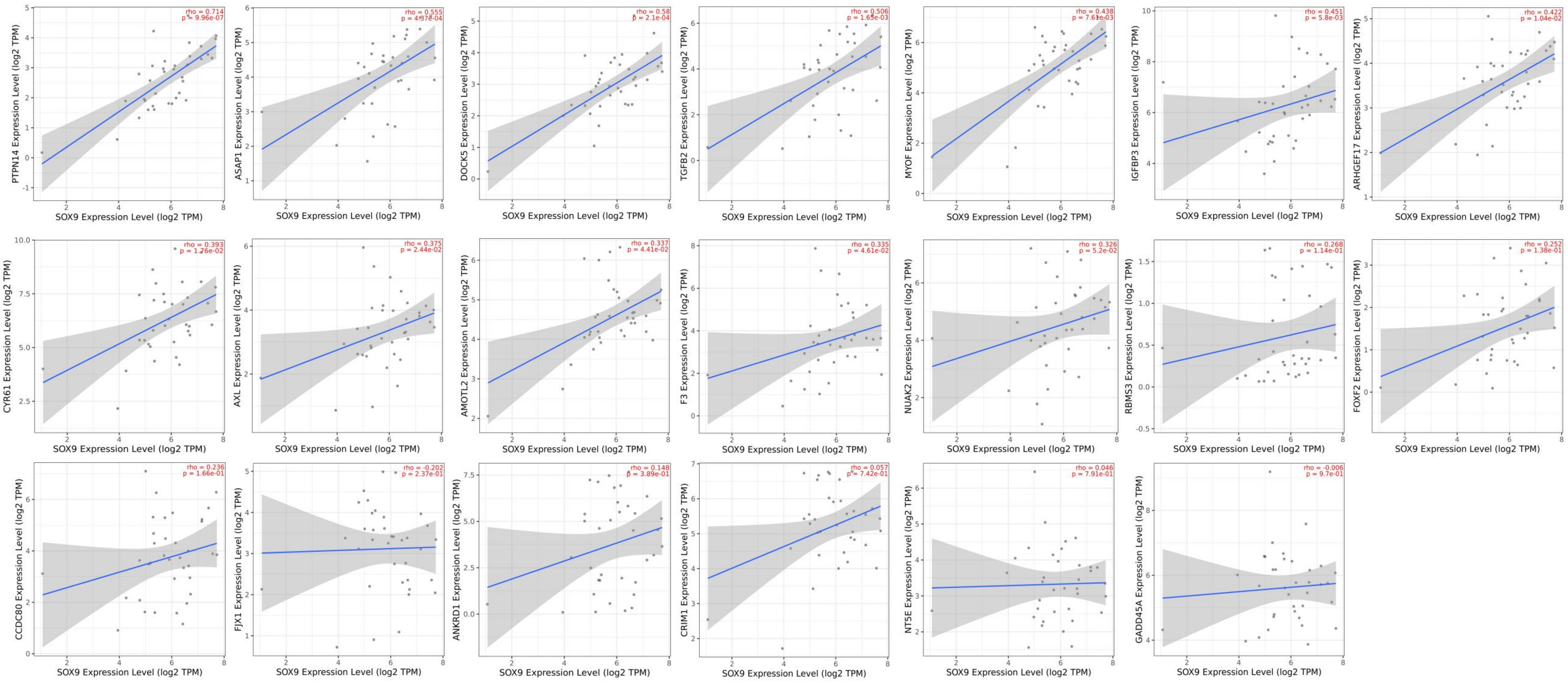

# Supplemental Figure S2

## S2C

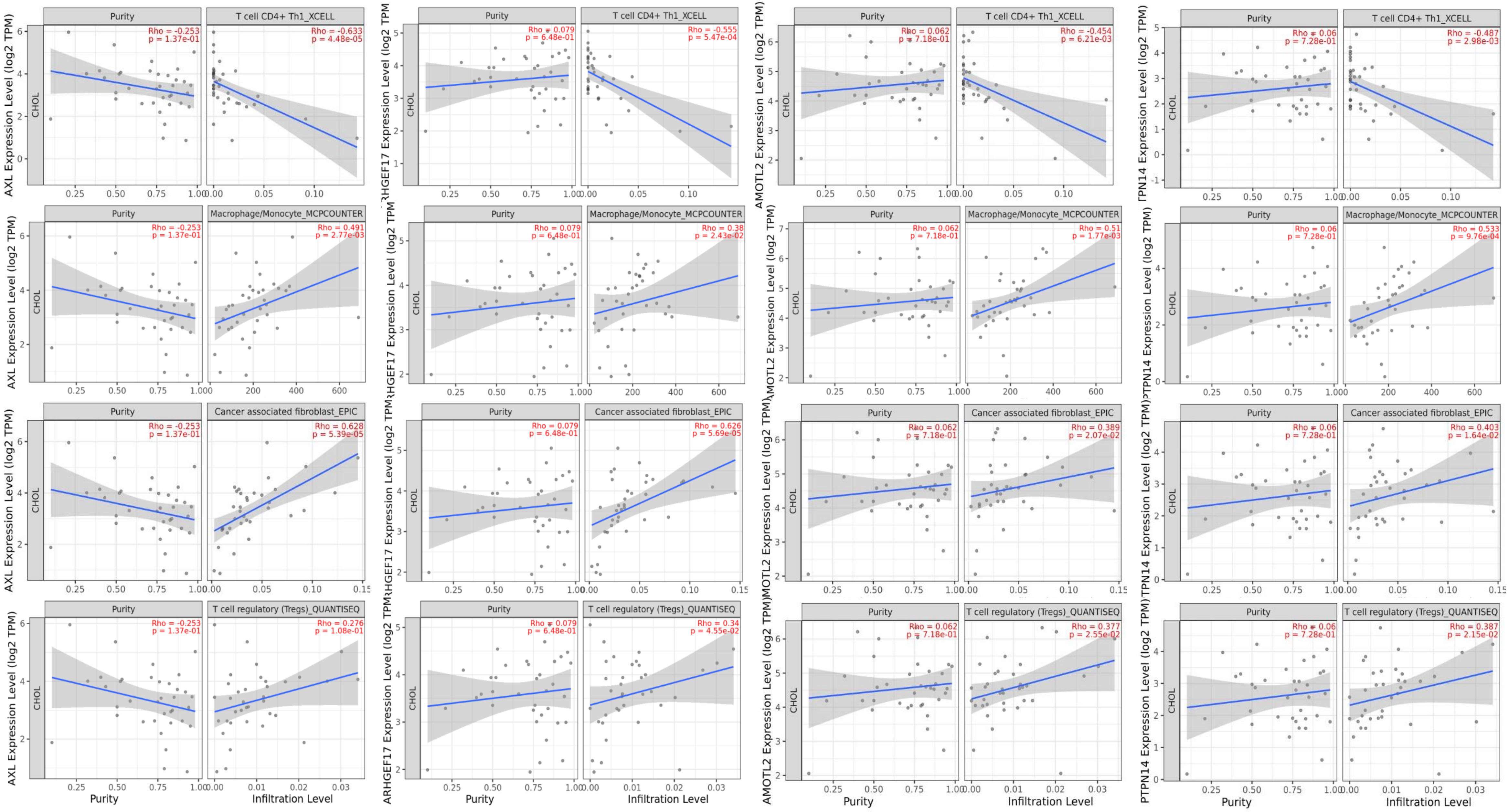

# Supplemental Figure S3

## S3A

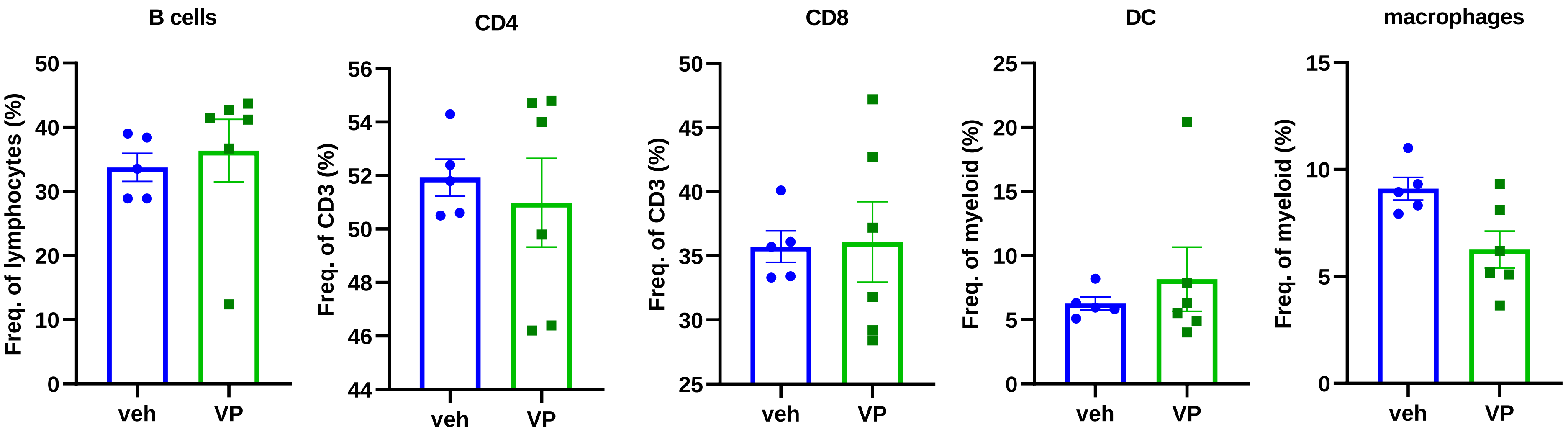


## S3B

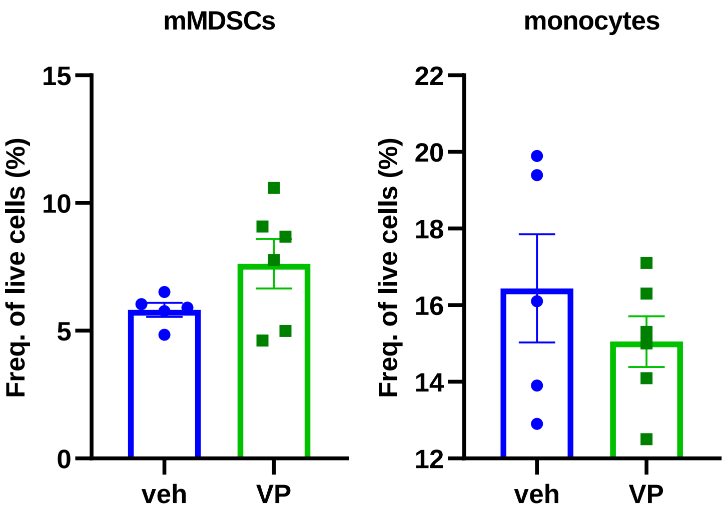

# Supplemental Figure S4

## S4A

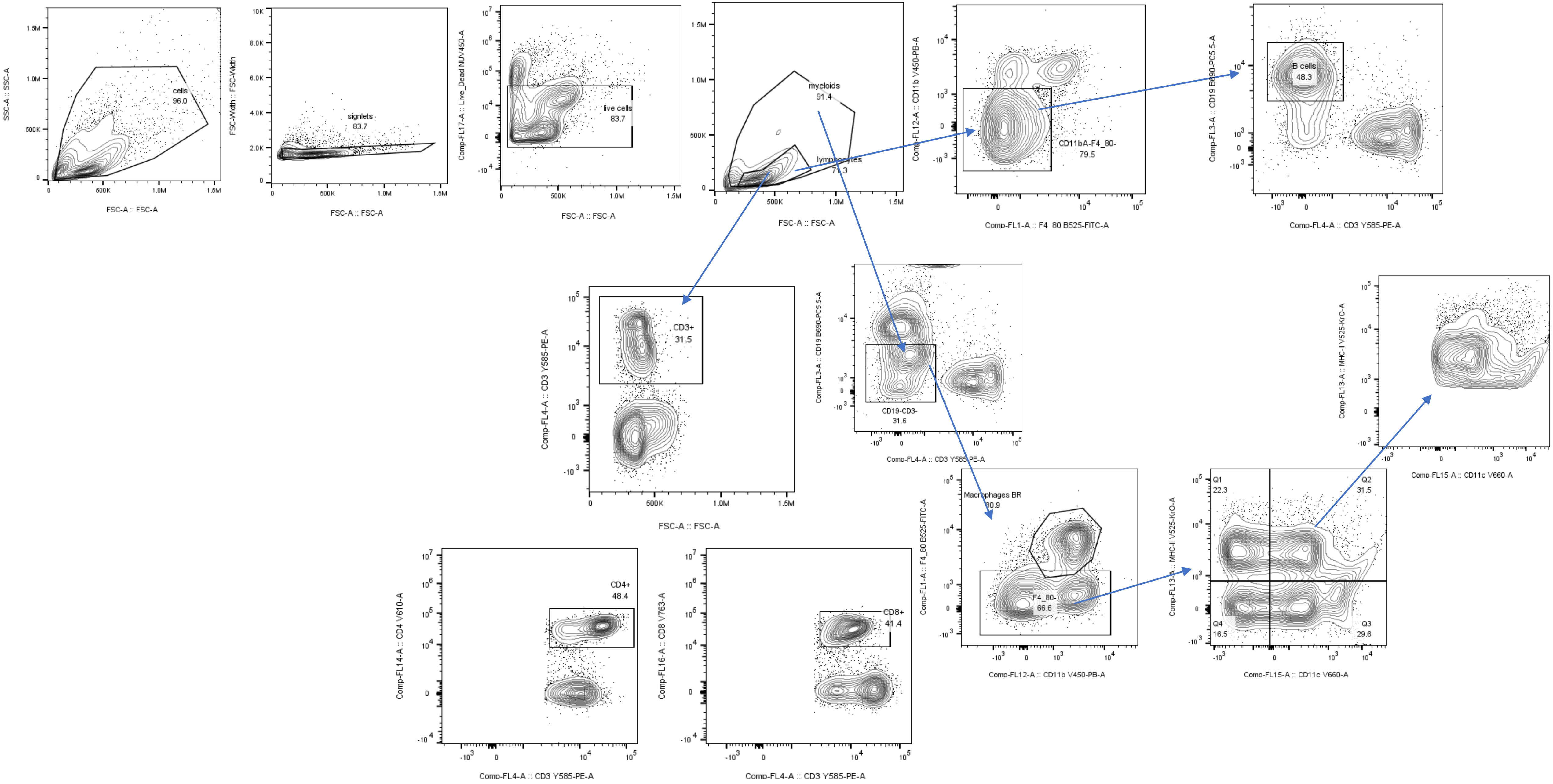

**S4B**

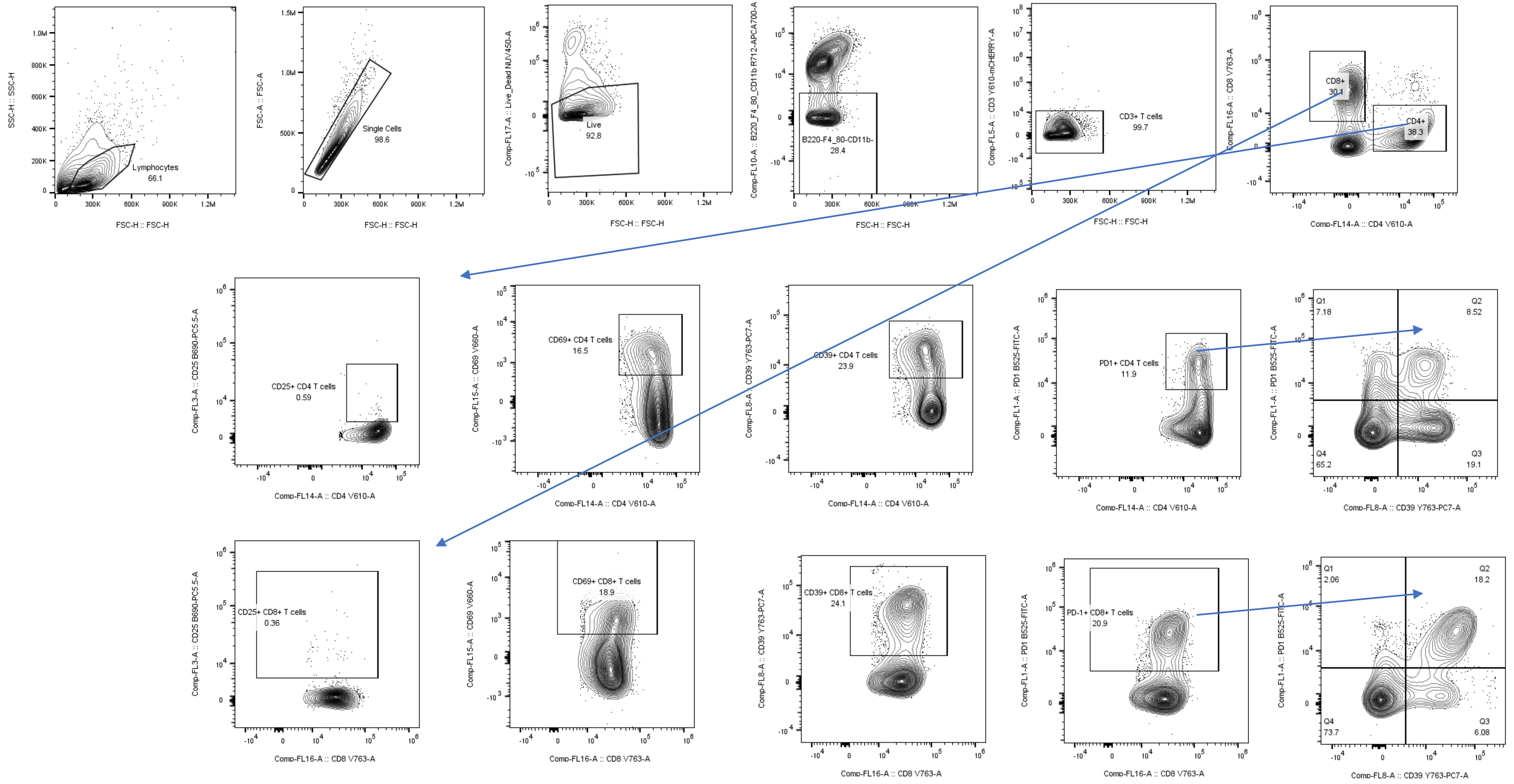
Lymphocytes 66.1
SSC-H :: SSC-H
FSC-H :: FSC-H
Single Cells 98.6
FSC-A :: FSC-A
Live 92.8
Comp-FL17-A :: Live_Dead NUV450-A
B220-F4_80-CD11b- 28.4
Comp-FL10-A :: B220_F4_80_CD11b R712-APCA700-A
CD3+ T cells 99.7
Comp-FL5-A :: CD3 Y610-mCHERRY-A
CD8+ 30.1
CD4+ 38.3
Comp-FL16-A :: CD8 V763-A
Comp-FL14-A :: CD4 V610-A
CD25+ CD4 T cells 0.59
Comp-FL3-A :: CD25 B690-PC5.5-A
CD69+ CD4 T cells 16.5
Comp-FL15-A :: CD69 V660-A
CD39+ CD4 T cells 23.9
Comp-FL8-A :: CD39 Y763-PC7-A
PD1+ CD4 T cells 11.9
Comp-FL1-A :: PD1 B525-FITC-A
Q1 7.18
Q2 8.52
Q4 65.2
Q3 19.1
Comp-FL8-A :: CD39 Y763-PC7-A
CD25+ CD8+ T cells 0.36
CD69+ CD8+ T cells 18.9
CD39+ CD8+ T cells 24.1
PD-1+ CD8+ T cells 20.9
Comp-FL16-A :: CD8 V763-A
Q1 2.06
Q2 18.2
Q4 73.7
Q3 6.08

**S4C**

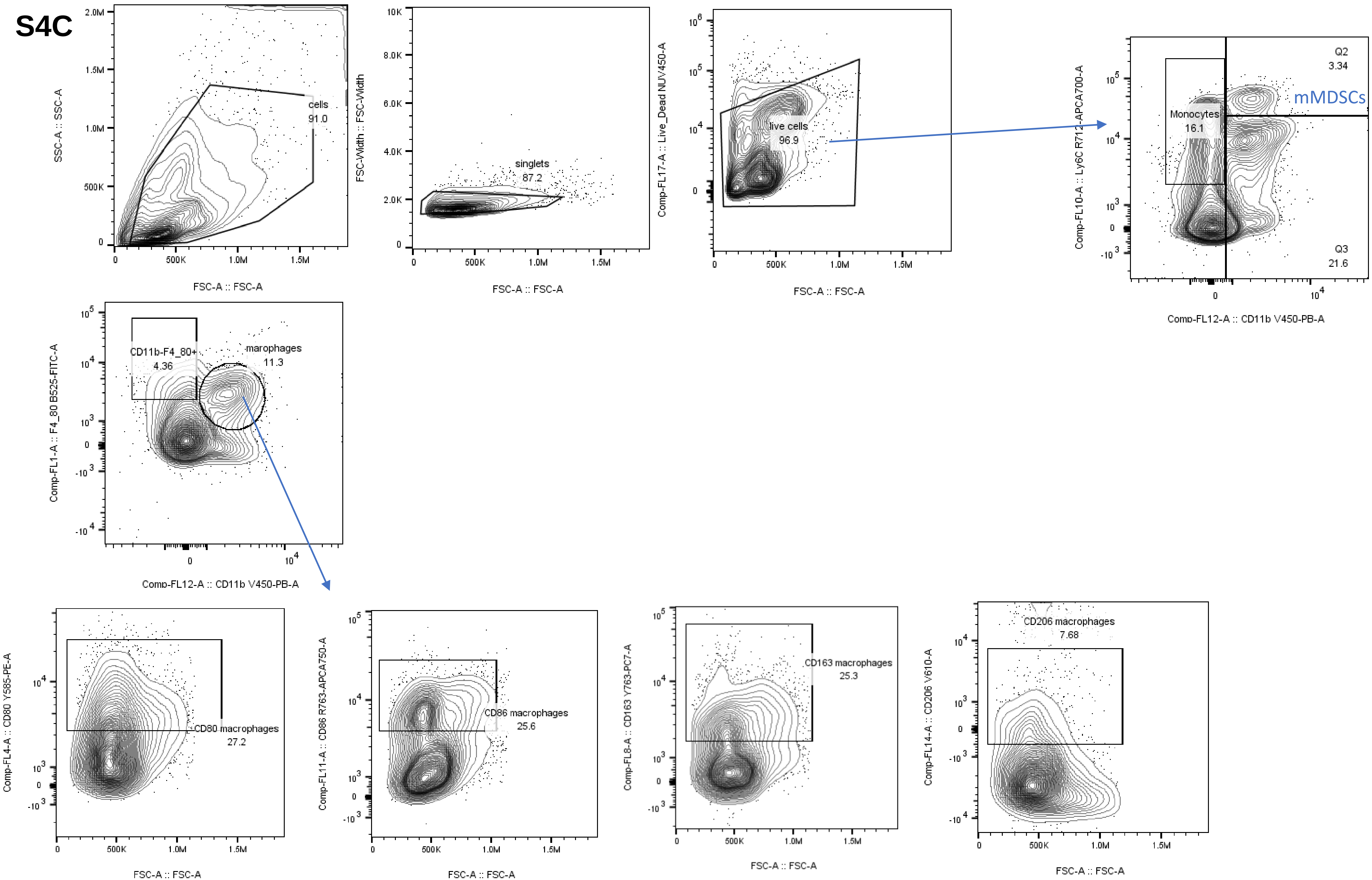

cells
91.0
SSC-A :: SSC-A
FSC-A :: FSC-A
singlets
87.2
FSC-Width :: FSC-Width
live cells
96.9
Comp-FL17-A :: Live_Dead NUV450-A
Monocytes
16.1
Q2
3.34
mMDSCs
Q3
21.6
Comp-FL10-A :: Ly6C R712-APCA700-A
Comp-FL12-A :: CD11b V450-PB-A
CD11b-F4_80+
4.36
marophages
11.3
Comp-FL1-A :: F4_80 B525-FITC-A
CD80 macrophages
27.2
Comp-FL4-A :: CD80 Y585-PE-A
CD86 macrophages
25.6
Comp-FL11-A :: CD86 R763-APCA750-A
CD163 macrophages
25.3
Comp-FL8-A :: CD163 Y763-PC7-A
CD206 macrophages
7.68
Comp-FL14-A :: CD206 V610-A

# Supplemental Figure S5
## S5A

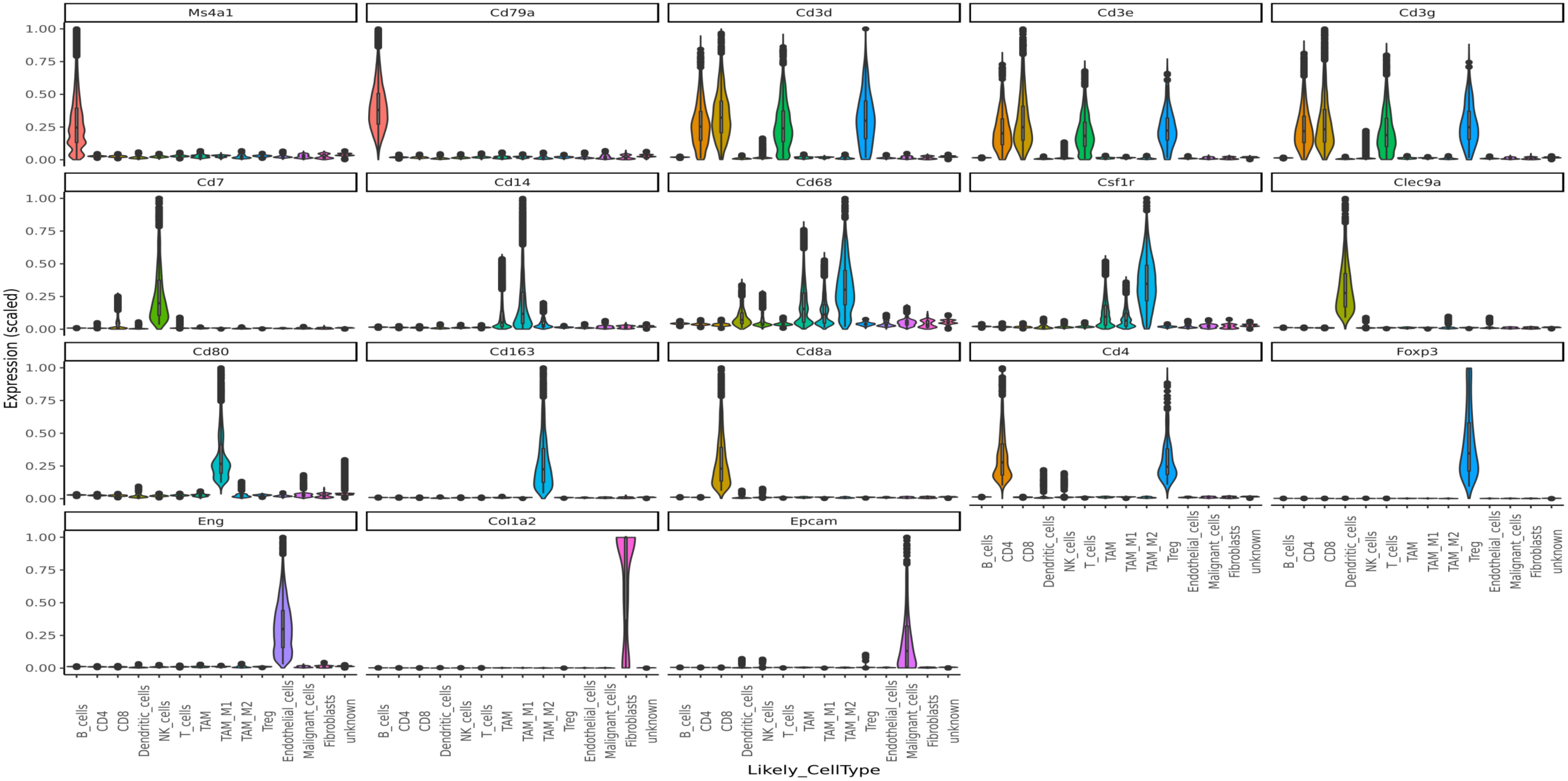

# Supplemental Figure S5

## S5B

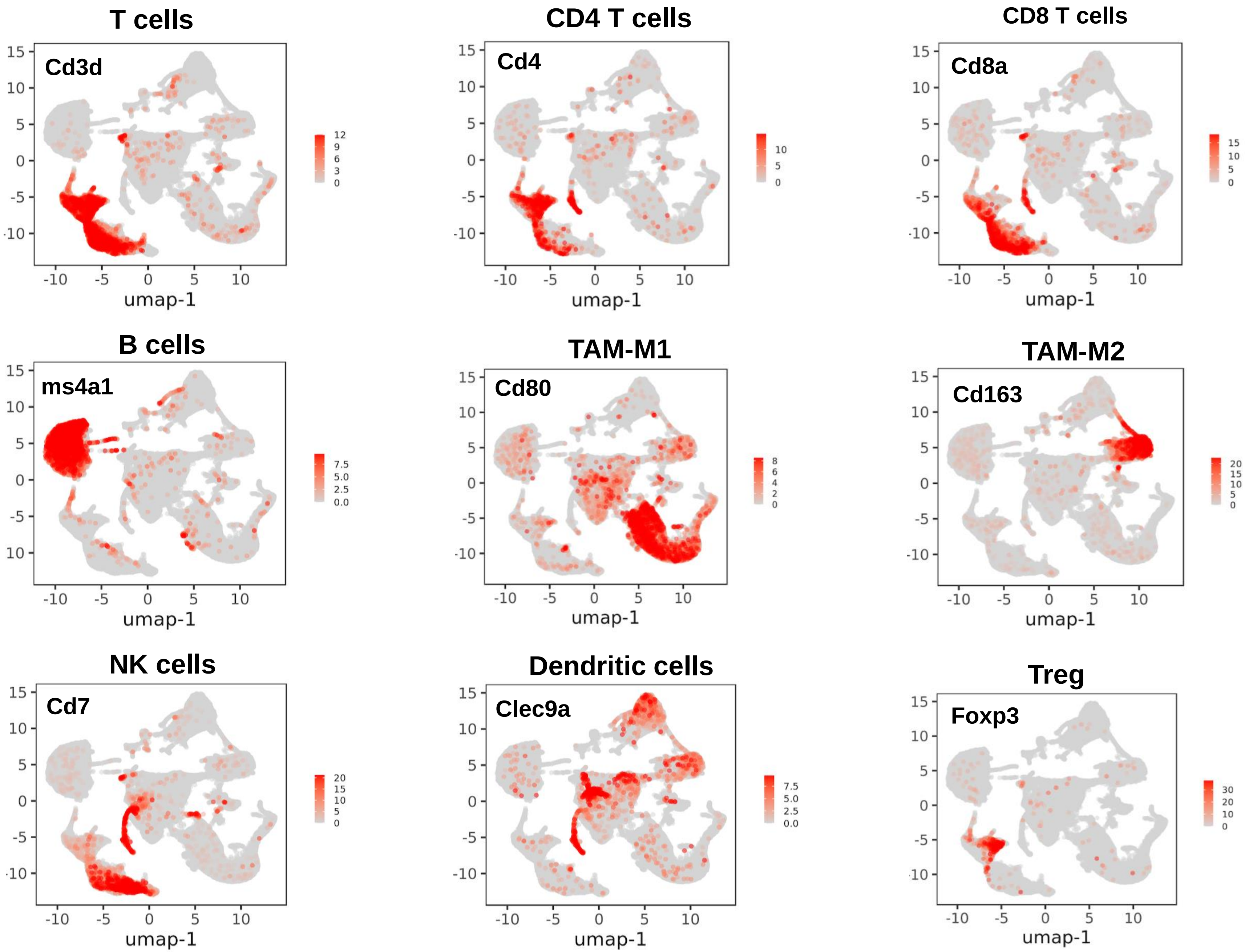

## Supplemental Figure S5

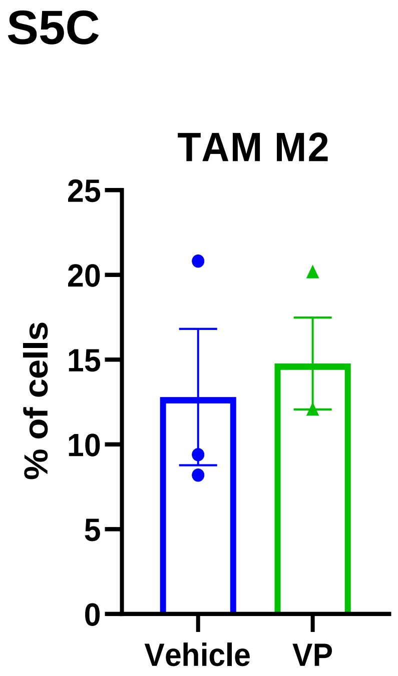


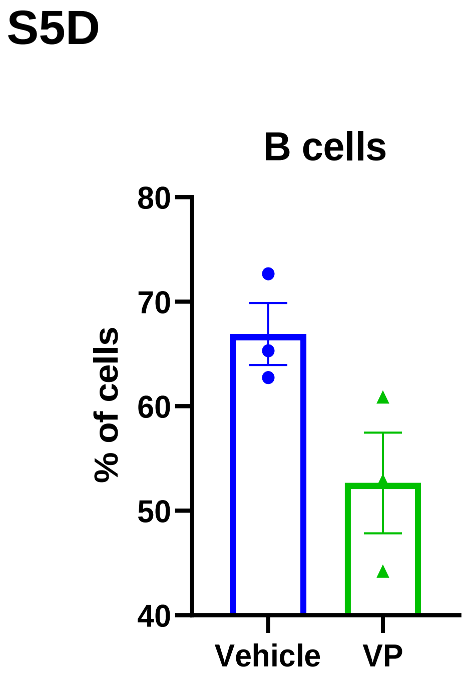


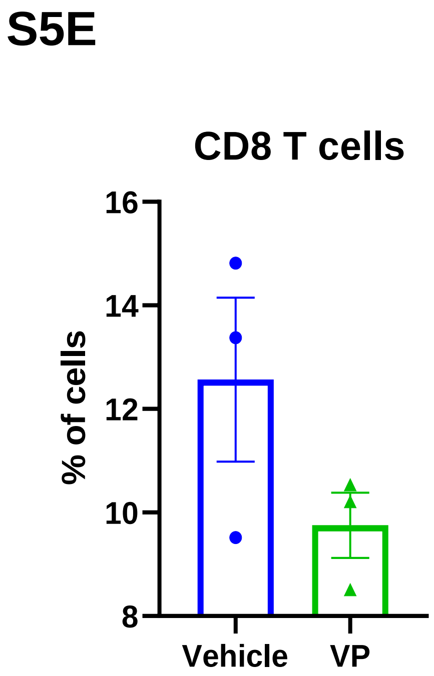

## Supplemental Figure S6

S6A

S6B

S6C

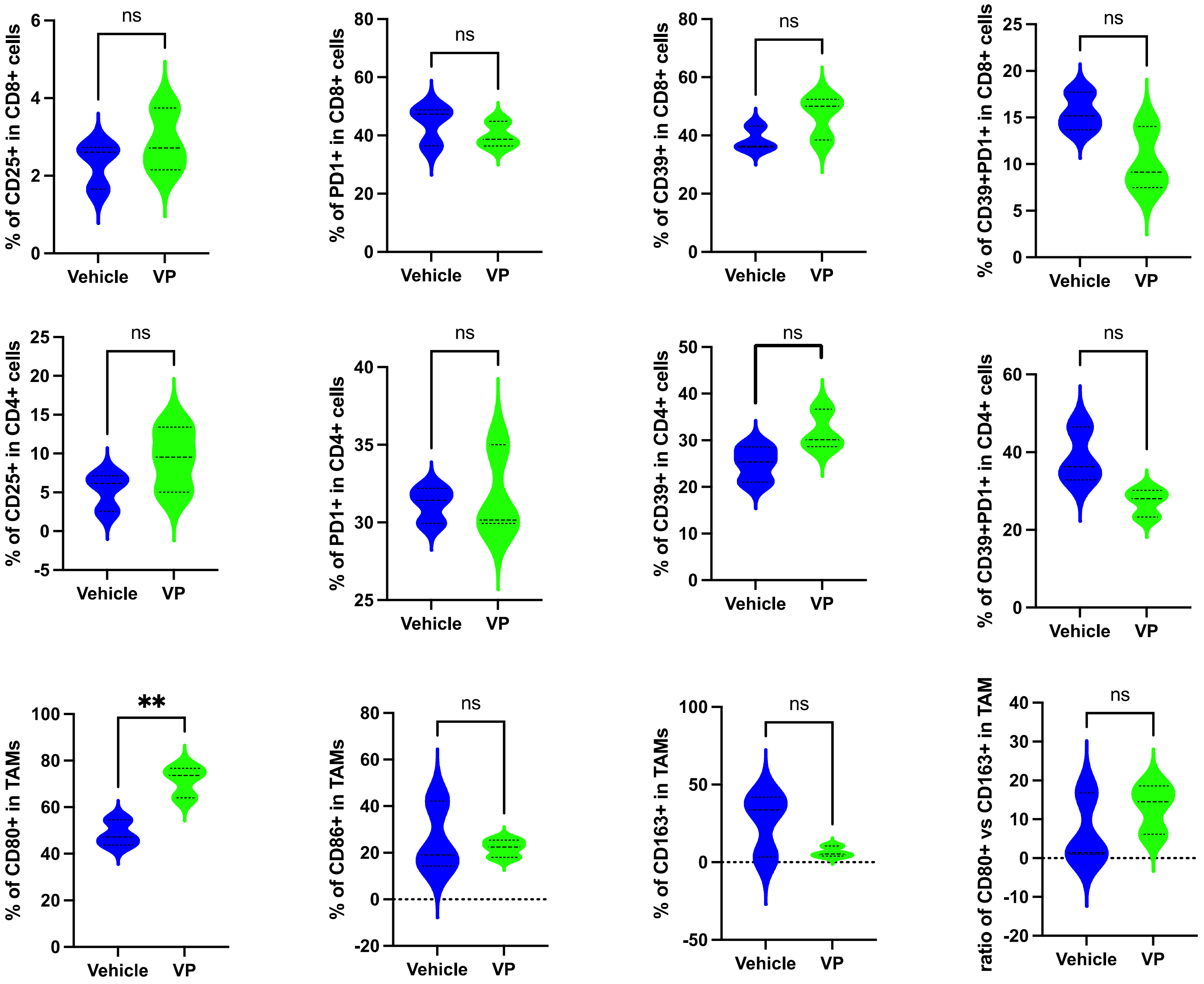

# Supplemental Figure S7

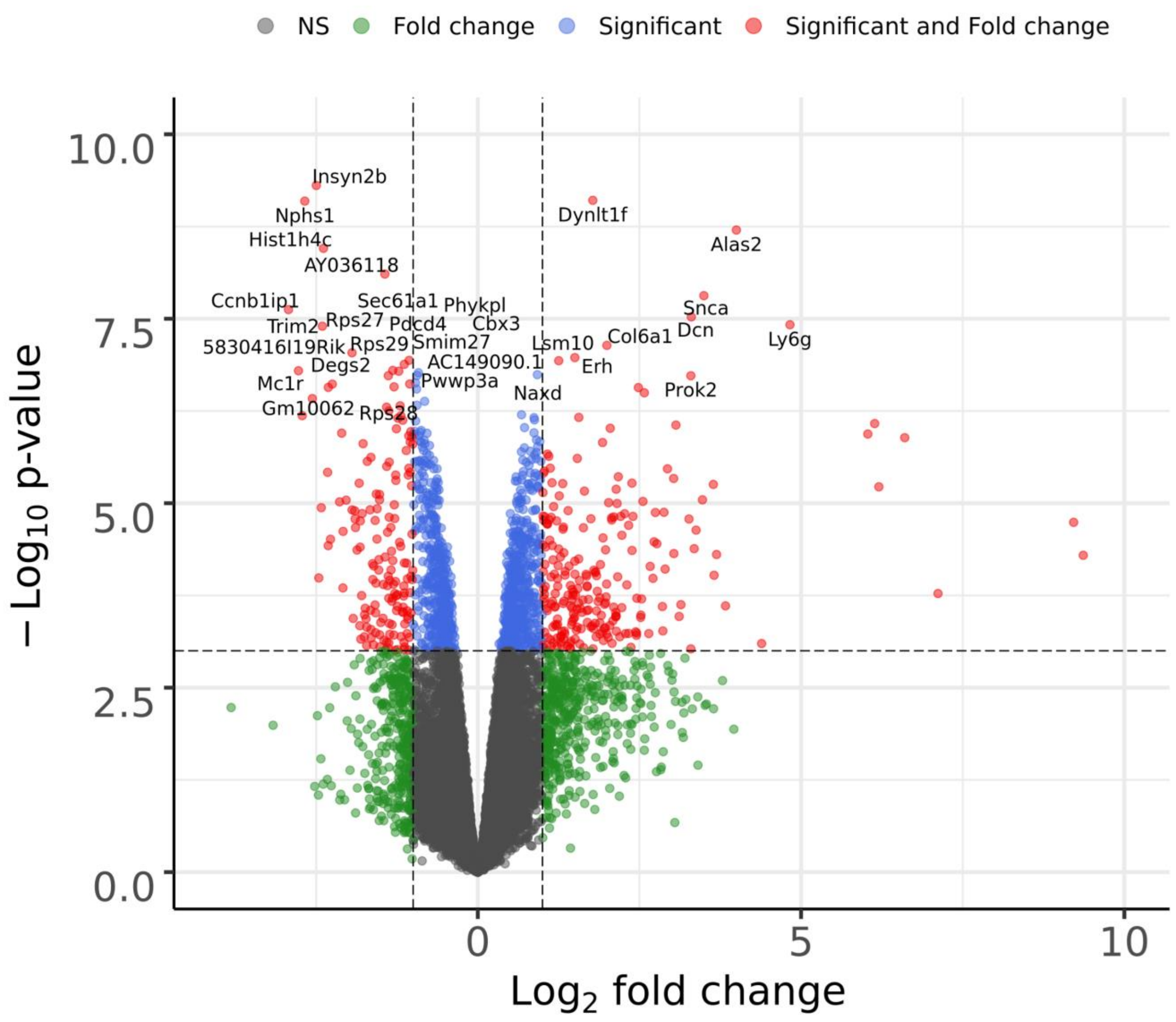

# Supplemental Figure S8

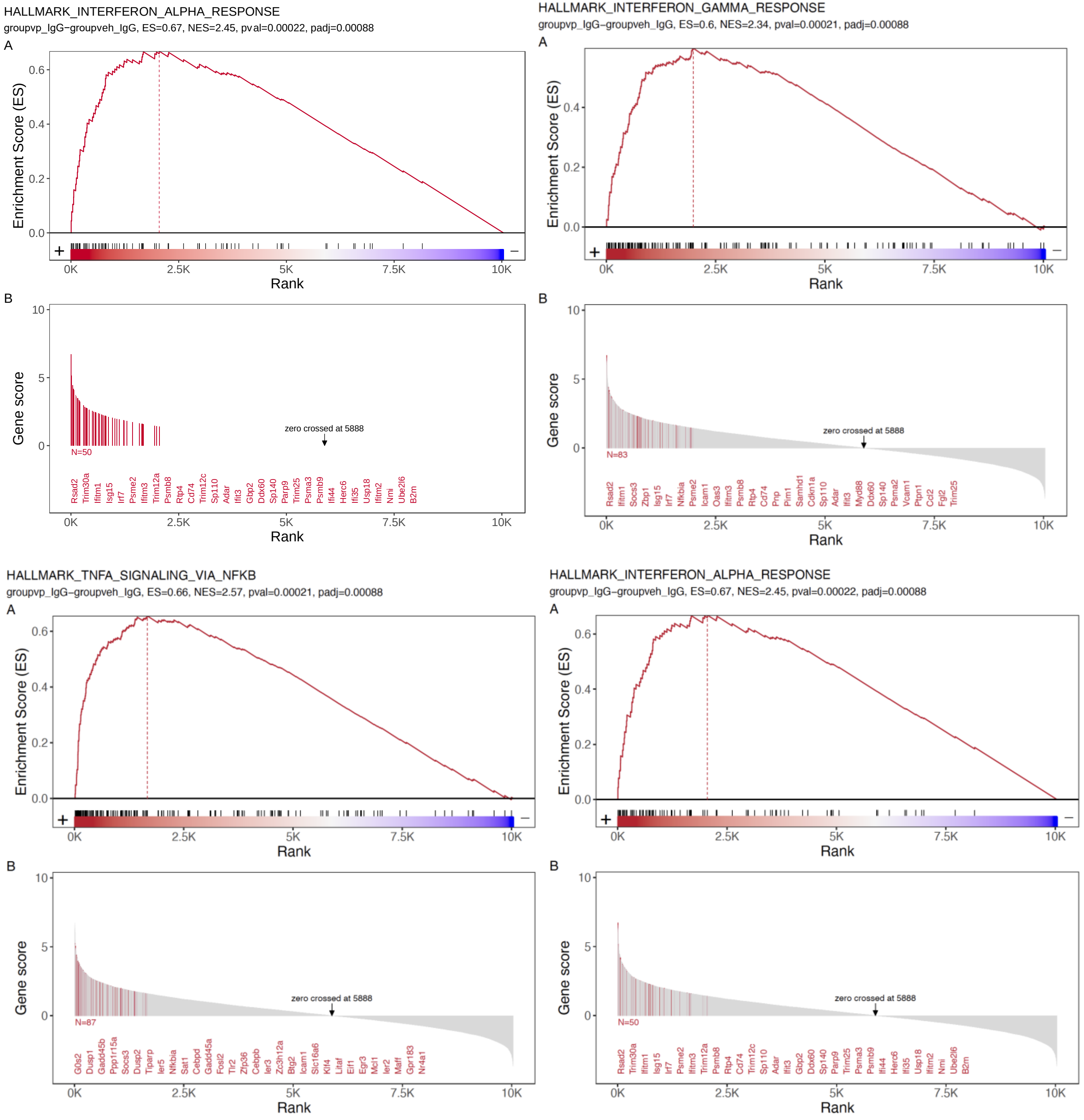

Supplement: Supplementary file 1 [file cancers-15-02454-s001.zip › Supplemental Figures.pdf]
